# Exploring life cycle and health-related food taboos and their associated reasons among Puntland - Somalia communities: a mixed methods cross sectional study

**DOI:** 10.1186/s40795-026-01413-1

**Published:** 2026-07-09

**Authors:** Hasifa Nanteza, Shukri Abdullahi Adam, Abdullahi Abdulkadir Mohamed, Marvin Joseph Senoga

**Affiliations:** 1 Department of Nutrition and Food Science, University of Bosaso, Garowe, Somalia; 2 Department of Geography, Geo-informatics and Climatic Sciences, Makerere University, Kampala, Uganda; 3 Faculty of Computing and Informatics, Makerere University Business School, Kampala, Uganda

**Keywords:** Food taboos, Life cycle, Health-related, Reasons, Communities, Puntland, Somalia

## Abstract

**Background:**

Food taboos are culturally rooted dietary restrictions that model food choices and many compromise nutritional status specifically among vulnerable populations. The study examined the prevalence of food taboos, tabooed foods and explored reasons for their existence among different segments of Puntland communities.

**Methods:**

A community based mixed methods cross-sectional study was conducted between September and December across the nine regions of Puntland state of Somalia. A total of 290 participants were selected using multi-stage sampling. Quantitative data were collected using structured questionnaires while qualitative data were obtained through 18 key informant interviews and 37 Focus Group Discussions (FGDs) involving pregnant and lactating women, elderly men and women, women of reproductive age and adult men. Data on demographic characteristics were analyzed using SPSS version 31, while qualitative data were analyzed thematically using systematic text condensation.

**Results:**

Overall, 88% of participants reported the existence of food taboos in their communities. Pregnancy and lactation were the life stages with the most restrictions (32%) and the least were during menstruation (12%). Commonly tabooed foods during pregnancy included honey, ghee, sheep meat, chili, papaya and whole grains primarily due to beliefs linking them to miscarriage, fetal deformities and labor complications. Liver was universally restricted for children under two years due to perceived speech delay. Men also faced prohibitions such organ meats except liver, animal head, neck and leg meat, considered foods for females and associated with shame, weakness and cowardice. Regional variations were observed with certain taboos being specific to regions, mostly Mudug and Eyl district of Nugaal region.

**Conclusion:**

Food taboos are common throughout Puntland but they are more rampant in Mudug region and Eyl district of Nugaal region. Some taboos reported during menstruation and illness have some scientific backup and may contribute to better management of these conditions. However, taboos like those reported during pregnancy and lactation may reduce dietary diversity and negatively impact overall nutrition and health outcomes in vulnerable groups.

**Supplementary Information:**

The online version contains supplementary material available at https://doi.org/10.1186/s40795-026-01413-1.

## Background

Food taboos are culturally rooted dietary restrictions that model food choices and many compromise nutritional status specifically among vulnerable populations [1]. They influence the acceptance or avoidance of particular foods, limiting the acquisition of sufficient macro and micronutrients [1, 2]. These taboos are mainly gender and age based, disproportionately affecting women and children compared to men and adults.

A number of studies conducted in Africa and Asia have reported pregnant women to be barred from consuming different foods. In Malaysia, pregnant women do not to eat cabbage, coconut and fish caught by anyone other than her husband [2]. Pregnant women cannot eat crabs, shrimp, squid, shellfish, moringa leaves, eggplant, snake fruit, pineapple, durian, and cassava in Indonesia [3]. On the other hand [1], reports restriction of foods such as meat products, fish, potatoes, fruits, beans, eggs, butternut and pumpkin in South Africa. In some parts of Uganda, pregnant women do not eat offals, chicken, wild birds, smoked meat and fish, sugarcane, garden egg, honey and fruits like oranges, mangoes and pineapples [4, 5]. Pregnant women in Ethiopia cannot consume foods that look whitish like eggs, fatty meat, and dairy products (milk, yogurt, cheese), sugarcane, vegetables, linseed, honey and fruits during pregnancy [6, 7]. A study done in Kenya reported restrictions on liver, intestines, kidney, milk, and eggs fearing to cause obstructed labor [8]. A study exploring knowledge of prohibited foods during pregnancy in Northern Tanzania identified fish, eggs, sweet foods, butter and milk from cows other than their own to as prohibited during pregnancy [9].

Research done in KwaZulu-Natal - South Africa reported restrictions for children under two years of age, specifically prohibiting salty foods. It noted the belief that salt adversely affects a child’s knees, perhaps delaying walking and motor development [10]. Additionally, the prohibition of some meals, believed detrimental to dental health and the immature digestive system contributes to dental decay and malnutrition [10, 11].

Research indicates that food taboos among adolescent girls in the regions of Singida, Dar es Salaam, and Kigoma prohibit the consumption of marbled lungfish, as it is believed that its consumption results in development of additional breasts [11].

In Papua New Guinea married young men do not eat fresh meat [12]. They can only eat smoked meat. Men should not also eat flatfish (soles and stingrays), snails and beans as they weaken the warrior’s strength and lead to stomach disorders [12]. On the other hand, the Akan men in Nigeria do not eat ripe plantain to avoid developing waist pains and taro (a species of cocoyam) else, they will fail to perform sexually [13].

Consumption of prohibited foods is believed to have varying effects ranging from health issues like miscarriage, abnormal fetus movement, complications during delivery [1], poor fetal development [11], the unborn child having a plastered body [7], preeclampsia, general body weakness, weight loss, being pregnant beyond the gestation period of nine months, bleeding and vaginal discharge [14, 15]. Researchers also report social effects (like social abandonment, public condemnation/punishment and bad omens) to those who partake in prohibited foods [11] and environmental effects like reduced sustainability of resources.

Foods mainly prohibited are energy foods mainly carbohydrates and proteins and micronutrient rich foods [3]. This prevents the acquisition of a balanced diet as it limits the quality and quantities of food consumed leading to significant nutritional deficiencies, including Iron deficiency anemia, Vitamin B12 deficiency, protein deficiencies among others [2] especially among the vulnerable groups, mainly pregnant and lactating women and children.

The prevalence of food taboos in Garowe communities remains significant alongside persistent challenges of conflict, climate change, and economic instability exacerbating food insecurity and malnutrition, which afflict millions of people in the country [16]. The intersection of food taboos with limited food availability and dietary diversity complicates efforts to improve nutritional status [17] in the region. In order to improve the nutritional status of people living in Puntland and reduce the levels of malnutrition and nutritional deficiencies, factors like food taboos that hinder the consumption of balanced diets and increase the risk of developing malnutrition should be studied and addressed.

Understanding food taboos is paramount in order to make recommendations that challenge/change various perspectives held by the Puntland community through development of educational campaigns and programs that create awareness and tackle these misconceptions. However, there is currently no documented information on food taboos in Puntland and Somalia at large, thus the need for this study.

This study was conducted to determine the prevalence of food taboos, identify prohibited food items among different stages of life/conditions, identify stages of life/conditions during which food taboos are observed and explore reasons for the existence of food taboos among Puntland communities.

## Methods

### Study area and setting

Puntland is located in the northeastern part of Somalia, with Garowe as its administrative capital and Bosaso as the commercial capital. It is composed of nine regions, including Bari, Nugal, Mudug, Sanaag, Sool, Cayn, Karkaar, Haylaan and Ra’as Aseir/Gardafuul. The state has an estimated population of approximately 4.9 million people [18] who share the same ethnicity, culture, language and religion (99% Muslim), spread over an area of 212,510 sq [18]. The region has a semi-arid climate characterized by hot temperatures and low, variable rainfall, which make it highly vulnerable to drought and food insecurity. Trade is the major livelihood activity, although a few people are involved in livestock farming (goats, sheep and camels).

### Research design

A community-based mixed methods cross-sectional study design was used to elicit information from participants using a structured questionnaire for demographic information and prevalence, Key Informant Interviews (KIIs) and Focus Group Discussions (FGDs) with 6 to 10 participants for in-depth responses from respondents. This study was conducted between September and December 2025.

### Sample size

We used the Cochran Formula [19] to calculate the sample size at an estimated prevalence of 50% (0.5), a 95% confidence level and a 5% margin of error.

$$n_{0}\;=\;(\mathrm{z}^{\wedge}2\;*\;\mathrm{p}\;*\;(1\;-\;\mathrm{p})\;/\;\mathrm{e}^{\wedge}2$$

Where:

*n*_0_ = required sample size.

Z = Z-value (1.96 for 95% confidence level).

p = estimated proportion of people with food taboos (0.5).

E = margin of error (0.05).

The sample size calculation was as follows.

$$n_{0}\;=\;(1.96)^{\wedge}2\;*\;0.5\;*\;(1\;-\;0.5)\;/\;(0.05)^{\wedge}2\;=\;384.16$$

The calculated sample size was 384 people. However, due to resource constraints, we reduced the sample to 290 people.

### Selection of study participants

We enrolled 290 participants into the study and numbers selected from each region depended on its size and population. Bari and Nugaal are the most populous therefore had 55 participants each, followed by Mudug 40 participants, Sool and Sanaag 30 participants each and 20 participants for each of the other regions. We purposefully selected one district from each region except Nugaal where we selected two (we wanted to cover the views of the people in the capital and at least another district) then randomly picked one to three rural and urban villages from that district.

### Participant sampling

#### Multi stage and stratified sampling

This method involves dividing the population into subgroups (strata) that share similar characteristics, and then selecting respondents from each stratum. For our study, the strata included pregnant women and lactating women, elderly men and women, women of reproductive age and adult men. We divided the population into these subgroups depending on their expected familiarity/knowledge of food taboos. The percentage of respondents selected from each stratum was as follows: Elderly 33%, pregnant/lactating women 33%, other women of reproductive age 22% and adult men 12%. This ensured a fair representation of each group in the study. We biased the selection of individual study participants more towards elderly people and pregnant/lactating women because they were believed to have more information about the subject. Information on number of pregnant/lactating women was obtained from the records of the respective health facilities where their FGDs were held in each region. Unlike most published studies that examine food taboos among women, our study sought to explore food taboos across the life span, looking at infants/children less than 2years, pregnant and lactating women and men plus food taboos during critical periods like illnesses and menstruation.

### Data collection

A structured questionnaire (supplementary file 1) was used to collect data on the demographic characteristics of participants and the prevalence of food taboos. An interview guide (supplementary file 2) and a Focus Group discussion (FGD) guide (supplementary file 3) with semi-structured questions were developed based on literature, tested and used to gather detailed views on the different foods considered taboos, the associated reasons, and what happened if one consumed a tabooed food. We sought in depth responses from 18 Key Informants including health professionals – mainly nutrition officers, midwives and nurses, and community and religious leaders and 37 Focus Group Discussions (FGDs) with 6 to 10 participants each.

We used semi-structured interviews and Focused Group Discussions because they help to capture the nuanced understandings and social contexts of food taboos from participants at different societal levels. The researchers developed the interview and FGD questions, pretested them on 15 participants, who were then excluded from the actual study. Researchers revised or excluded unclear questions, those that received poor responses, or were not culturally appropriate to ensure rich, meaningful, and comfortable data collection. They modified the interview guides whenever gaps in information became apparent during the data collection process.

The research team conducted interviews with health professionals at health facilities and interviewed other participants at their homes or workplaces. FGDs with pregnant/lactating women were also done at health facilities while other participants were mobilized and gathered by community leaders at convenient facilities like educational centers. Researchers and assistants trained prior to the commencement of the data collection exercise did this. All answers were audio recorded and transcribed to allow better synthesis. Interviews lasted 30–60 min. Research assistants took field notes and captured key group dynamics like general group mood, dominant speakers and non-verbal cues during FGDs.

We held 37 FGDs, six in Bari and Nugaal, five in Mudug, four in Sool and Sanaag, and 3 in each of the rest of the regions. The researchers determined the necessary information and then sought out knowledgeable or experienced individuals willing to share it [3]. These included elders, pregnant/lactating women, women of reproductive age and adult men. FGDs for the elders combined both elderly men and elderly women. Each FGD took between 50 and 90 min.

Data underwent a rigorous post-collection review to identify and correct any inconsistencies or errors, thereby validating its quality.

Only open-ended questions were asked in the FGDs, with new questions developing from the feedback provided. Participants had the opportunity to expand upon suggestions and iterate on concepts raised by others.

### Data analysis

Data on demographic characteristics was analyzed using statistical package for social sciences (SPSS) version 31 to obtain descriptive statistics. Field notes and voice recordings taken during the interviews and focus group discussions were synthesized, data was manually analyzed, applying verbatim and principles of systemic text condensation [20]. The audio recordings were repeatedly listened to and transcribed notes reviewed to gain deep understanding of the overall content. Researchers did multiple checks during data analysis to ensure a deep understanding and accurate interpretation of the transcripts. The necessary meanings were extracted, summarized using a color-coding system, then grouped into major thematic categories. The thematic categories that emerged included pregnancy, lactation, infancy/early childhood, menstruation, adulthood (men) and illness.

## Results

### Prevalence of food taboos and social demographic characteristics of participants

Data on prevalence and demographic characteristics of participants was collected using a structured questionnaire administered to 290 respondents and 284 of them participated in FGDs, giving a response rate of 98%. Of these, 255 (88%) reported the existence of food taboos in their communities. Majority of the participants were females 181 (62%) and their ages ranged between 20 to above 50years. One hundred thirty eight (47.8%) participants were married, the rest were either single (38.8%), widowed (6.8%) or divorced (6.6%). Most respondents had no formal education 78 (26.8%). The rest (23.8%, 26.4% and 23%) had acquired primary, secondary or college/university education respectively, Table 1.

**Table 1:** Sociodemographic characteristics of participants

Participants’s characteristic	Category	Number	Percentage (*n* = 290)
Gender	Male	110	38
	Female	181	62
Age range	20–30	54	18.6
	31–40	59	20.2
	41–50	73	25.2
	Above 50	104	36
Education level	Informal Education	78	26.8
	Primary Level	69	23.8
	Secondary Level	76	26.4
	College/University Level	67	23
Marital status	Single	112	38.8
	Married	138	47.8
	Divorced	18	6.6
	Widowed	22	6.8

### Stages of life/conditions during which food taboos are observed

**Fig. 1:**
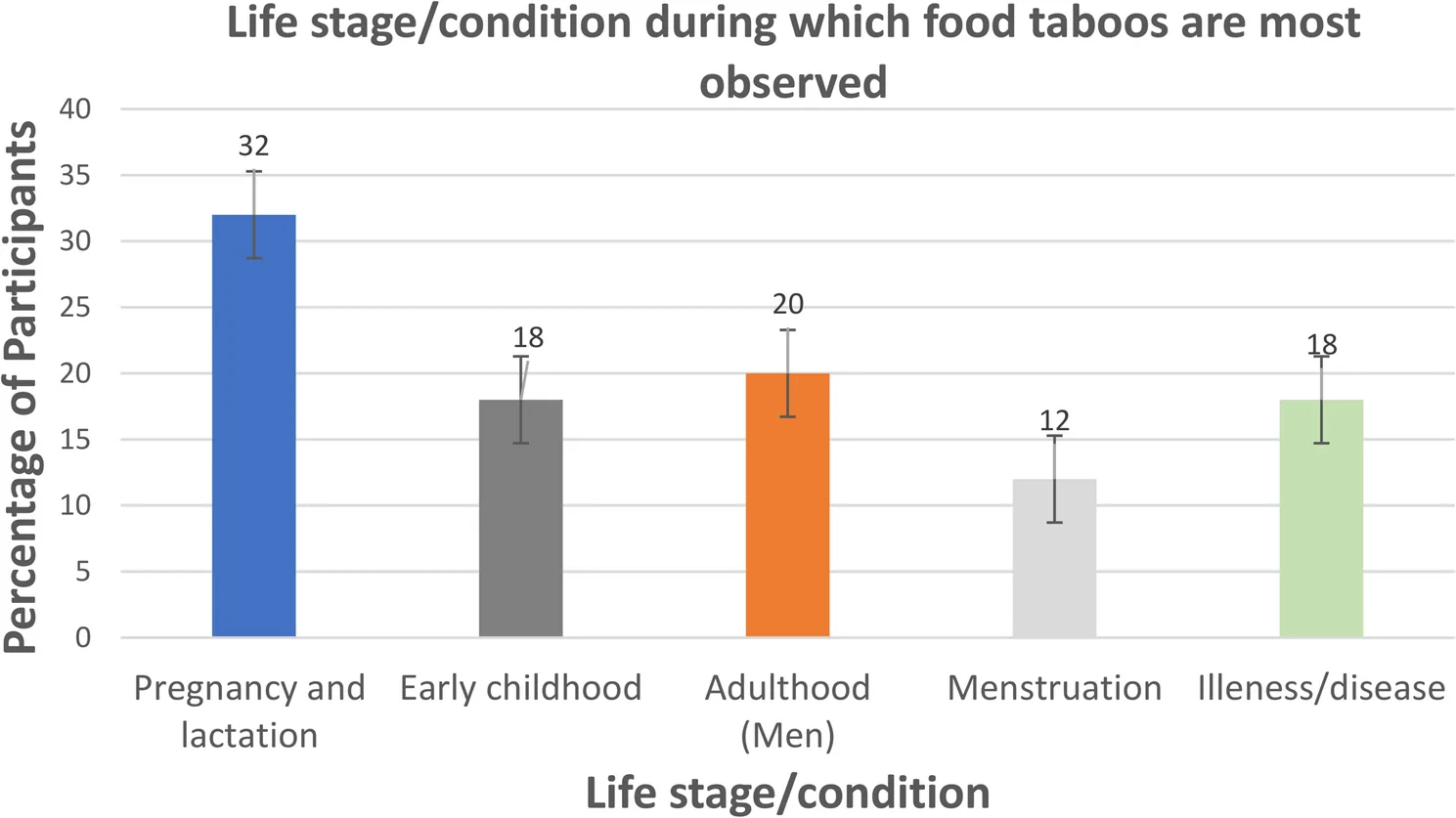
Stages of life during which food taboos are most observed

Among the 255 respondents who reported taboos, the life stage with the most restricted food was pregnancy and lactation, 80 (32%) of respondents reporting food restrictions during these periods. The lowest number of restrictions was reported during menstruation, at 30 (12%) as indicated in Fig. 1.

### Food taboos during pregnancy

This is the life cycle stage with the most food restrictions. These are based on cultural, health, or traditional beliefs aimed at “protecting” both the mother and her child. We found restrictions for nine foods across all regions including honey, ghee and sheep fat, meat, papaya, cantaloupe, whole grains, chili, and lemon. Dates, fish, eggs, preserved meat, fenugreek (xulbad) liver, kidney and having large meals in the third trimester (Table 2) were prohibited in some regions. Some participants reported different experiences with these foods:

**Table 2:** Prohibited food items among pregnant women in Puntland - Somalia

Prohibited food	Reason(s) for prohibition	Region/district
Chili	- Absence of hair in newborns - Baby is born with red eyes	All
Honey	Miscarriage	All
Ghee and sheep fat	Miscarriage	All
Papaya, lemon and cantaloupe	- Acne to the unborn baby - Stomach cramps and diarrhoea to the mother	All
Sheep meat	Miscarriage	All
Whole grains	Complications during labor and delivery.	All
Coffee	Miscarriage	All
Dates	Miscarriage in the first trimester	Nugaal, Mudug
Fish	- Nausea and vomiting in the first trimester - Delays teething - skin rash to the unborn baby	Nugaal (Eyl), Mudug
Cold drinks	-Causes constipation -Big sized baby, difficult to deliver	Nugaal
Eggs	Baby will be bald	Nugaal
Preserved meat	Spontaneous abortion/miscarriage	Sanaag, Sool, Haylaan
Liver and kidney (during first trimester)	Causes structural deformity in unborn baby	Mudug and Nugaal (Eyl)
Having large meals in the third trimester	Excess sized baby, causing rapturing during normal birth or caesarean section	Nugaal (Eyl)
Fenugreek (xulbad)	Miscarriage	Nugaal

One FGD participant (a pregnant woman, Sanaag) stated, “*I will never repeat the same mistake again! I lost my first pregnancy because I took honey. I was two months pregnant and took honey for a common cold that I had developed. In the evening*,* I started bleeding and lost my first baby just like that. When I told my mother about it*,* she said*,* “oh my child! You killed your own baby”. Honey should not be taken during the first trimester because it causes miscarriage”.*

Another one from Mudug said, “*My sister got a miscarriage after adding sheep fat to her dinner. We always add it to digir (a common meal from red beans commonly consumed for dinner among the Puntland people) but not for pregnant women. My sister had recently returned to the country and she did not believe in our traditions so she added the fat to her meal*,* in the next few days*,* she lost her baby”.*

On the contrary, a KII participant (religious leader from Garowe) said, *“We are Muslims and our prophet Mohammad (peace be upon Him) should have told us that honey is not good for pregnant women*,* but he did not say anything about it. Instead*,* he encouraged it for its medicinal benefits though not out rightly to pregnant women. I think it goes back to the concept of extravagancy*,* for even water can be toxic if consumed in unacceptably large amounts. Consuming a reasonable amount of honey cannot cause a miscarriage. I honestly believe that this is a misconception because I have lived with non-Somali people who use honey during pregnancy without having any miscarriages”*.

A KII participant from Ra’as Aseir who identified herself as a midwife recounted: …. *in my 22-year career as a midwife*,* I have come across babies with deformed lips*,* ears or limbs. I have advised the mothers to avoid meat and papaya and cantaloupe in their next pregnancies. They are responsible for these deformities.*

Some foods high in vitamins A and C are also prohibited. These include chili, papaya, lemon and cantaloupe. Chili is believed to cause absence of hair in newborns and the baby being born with red eyes. On the other hand, papaya, lemon and cantaloupe are believed to cause stomach cramps and diarrhea to the mother and acne to the unborn baby.

An elderly KII participant (a community leader from Bari) reported “….*chili is liked by most pregnant women because it helps to manage nausea and vomiting and improves their appetite. Nevertheless*,* they have to avoid it to prevent absence of hair in newborns and the baby being born with red eyes. The child is rebuked and teased by other children as they grow up. Their beauty would also be highly compromised*,* making it difficult to find a husband or wife in future”.*

Another, KII (a nutrition officer from Garowe – Nugaal) told us, *“When I was pregnant with my first child*,* I went to my antenatal appointments to X (a very famous midwife in Garowe district). At the end of each appointment*,* she reminded me not to eat papaya and cantaloupe (locally known as batiiq) because they would cause acne to the baby. However*,* because of my knowledge of nutrition and the benefits of these fruits*,* I went ahead and ate them during pregnancy. My child was born with fine skin”.*

### Lactation

The only foods restricted in all regions were spicy foods and black tea. These are believed to cause diarrhoea and stomach pain to the breastfeeding baby, change the taste of milk and prevent secretion of enough breast milk respectively. Other regional food prohibitions are in Table 3.

**Table 3:** Prohibited food items among lactating women in Puntland - Somalia

Prohibited food	Reason(s) for prohibition	Region/district reporting food taboo
Spicy foods	-Cause diarrhoea and stomach pain to the breastfeeding baby -Changes the taste of milk	All
Black tea	-Prevents secretion of enough breast milk	All
Carbonated beverages	-Cause stomach pain, dehydration and hiccups to the baby - Heartburn to the mother	Nugaal
Coffee	- Sleeplessness - Damages the baby’s brain	Mudug and Nugaal (Eyl)
Rice and spaghetti	-Cause constipation -Reduce breast milk production	Bari, Karkaar

### Infancy and early childhood

We asked participants about foods considered a taboo to children aged 6 months to 2 years. All participants unanimously reported liver not to be given to children before 1 year as it causes speech delays. Eggs, fish, lemon, beans, maize, sorghum, wheat, banana, chocolate and chili are taboos in some regions, mainly Mudug and Eyl district, as shown in Table 4. Although lemon is a taboo in Mudug because it over sharpens the baby’s teeth causing them to bite everyone, it is encouraged to be given to children in Sanaag and Sool for early teething and strong teeth formation.

**Table 4:** Prohibited food items among children 6 months to 2 years in Puntland

Prohibited food	Reason(s) for prohibition	Region/district reporting food taboo
Liver	-Delays speech, high amounts of vitamin A that cause health issues to the baby.	All
Eggs	-Causes blisters to the skin	Nugaal/Eyl
Fish	- Cannot be digested by children less than 2yrs - Causes choking (bones)	Nugaal/Eyl
Lemon	-The teeth become too sharp and bite others	Mudug
Beans	-Cause diarrhoea -Cause infections like tonsillitis	Nugaal/Eyl and Bari
Maize, sorghum, wheat	-Difficult to chew	Bari
Banana	-Causes diarrhoea	Mudug
Chocolate	-Speech delay	Karkaar
Chili	-Irritates child’s mouth	Nugaal

*“…. We force young children to take pure lemon juice without sugar…… It helps their teeth to come early and strong”. (FGD participants from Sanaag and Sool).*

Another participant, a pregnant woman from Bari said, *“We do not give our children beans. They give the child diarrhea and infections like tonsillitis”.*

Another FGD participant from Karkaar said, *“I have six children and I have never fed any of them liver before they reach one year. Liver delays speech.* Another one added,

*“I learnt my lesson and I no longer give eggs to my children before they are one. I gave eggs to my first-born daughter and she would get blisters on her skin especially the limbs. We went to different doctors and specialists even outside town but they could not tell the cause. A dermatologist in Hargeisa told us to stop giving her eggs and that was the end of it*”. (FGD Lactating woman from Eyl district).

### Adulthood (Men)

Unlike most cultures all over the world, which may not present any food taboos for men, the Somali culture is unique, prohibiting some foods for adult men. These include organ meats except liver (intestines, heart, and kidney), head, neck and leg meat of any animal, porridge, leftover food and chocolate in some areas like Mudug and Eyl district (Table 5).

**Table 5:** Prohibited food items among men in Puntland – Somalia

Prohibited food	Reason(s) for prohibition	Region/district
Intestines (caloole), kidney, head and leg meat	-Considered as food for women -It is a shame -People will disrespect you	All
Animal heart	-Causes cowardice	All
Neck meat	-The man’s neck becomes weak and bends	All
Chocolate	-It is a shame	Mudug, Nugaal (Eyl)
Porridge	-Considered to be food for lactating women	Mudug, Bari
Leftover food	-Considered to be food for females	Mudug
Leefleefow ( a local sauce)	-Considered to be food for females	Mudug

A male KII (a community leader from Haylaan) told us, “… *men do not eat organ meats except liver (beer) of any animal. They do not eat animal head*,* neck and leg meat. These are all considered foods for females and bring great shame to any man who indulges in them”.*

Another one from Cayn said, *“….eating the heart causes cowardice in men. If a man eats meat of the neck of an animal*,* his neck weakens and it bends sideways. Everyone in the community will then know about what he did and stigmatize him”.*

A KII nutrition officer from Bari said, “*Our ancestors were very wise. They wanted to protect their daughters from stinginess of their husbands. Organ meats except liver*,* are all poor quality meat and they are very cheap compared to real meat. If men were able to eat these*,* they would not give the women money to buy good/high quality meat*,* which is expensive. By banning them from eating these kinds of meat*,* they are bound to buy good quality meat that the whole family can enjoy.*

However, a FGD participant from Mudug stated, “*We do not eat offals at home*,* but we eat in the market (restaurants). I personally enjoy it so much. I am not yet married but I will continue to enjoy it at the market restaurant even when I get married; but I will not eat it at home to preserve my respect as a man in the home”.*

Another FGD participant said: *Liver is a high profile food. It is served to those with high status. If a household prepares liver for example*,* it is served to the husband who eats to his full then gives the leftover to rest of the family.* He also added that *if someone slaughters an animal*,* (a goat*,* sheep a camel) for a function/festival*,* the liver is given to the high profile guests with high positions in society like the sheikhs (Islamic religious leaders)*,* politicians*,* successful businessmen among others.*

An elderly KII from Mudug made an interesting submission. *“…not a taboo but men from Mudug eat fish while closing their nose. We hate the smell of fish and we therefore do not like it much. In addition*,* we do not eat chicken in Mudug. We do not have any chicken farms in our region and there is no chicken trade. Our people therefore do not know chicken”.*

Another participant emphasised *“. I only tasted chicken for the first time when I went to Asia for my master’s degree studies. Chicken is one of the commonest foods there and I had very limited options*,* so I tried it”.*

### Menstruation

The commonest restriction among all Puntland communities is cold drinks especially ice and milk tea. Cold drinks are believed to slow menstrual flow, increase menstrual cramps/pain and contribute to future ill health, while milk tea contributes to weight gain and causes enlargement of breasts and harms virginity. Other restricted foods are coffee, spicy food and fenugreek as shown in Table 6. These are particular to specific regions and are related to health issues.

**Table 6:** Prohibited food items among menstruating women in Puntland - Somalia

Prohibited food	Reason(s) for prohibition	Region/district
Cold drinks especially ice	- Slow menstrual flow -Increase menstrual cramps/pain (dysmenorrhea) - Future ill-health	All
Milk tea^a^	- Makes them fat -Causes enlargement of breasts and harms virginity	All
Coffee	- Worsens cramps	Mudug, Karkaar
Spicy food	-Causes diarrhoea	Mudug
Fenugreek	-Heavy bleeding	Nugaal

^a^ Milk tea is prohibited for all young women

A FGD participant (young woman from Nugaal) told us *“I get a lot of back pain if I take cold drinks during my periods”.* On the contrary, another FGD participant from Haylaan stated, *“I eat all those foods and my periods are always peaceful and normal. I have never experienced any of those issues”.*

### Illness/disease

In Puntland, food taboos significantly influence the incidence and management of specific illnesses such as diarrhea, gall bladder disease and fractures. In the case of diarrhea, food taboos exclude mango juice, milk, vegetables and spices because they worsen the condition by speeding up bowel movements, irritating the intestinal lining and exacerbating symptoms. Acidic foods especially citrus fruits, chili and foods with a lot of fat are believed to cause heartburn and worsen the condition of peptic ulcer patients. In addition, ghee and sheep fat are believed to interact with medications rendering them ineffective. On the other hand, sugary foods (like tea, juice, cake), fish and beans are believed to increase injury, delay healing or cause infection in people with fractures respectively (Table 7).

**Table 7:** Prohibited food items among people with an illness in Puntland - Somalia

Prohibited food	Reason(s) for prohibition	Region/district
Ghee and sheep fat	-Interacts with medication rendering it ineffective	All
Acidic foods and chili	-Cause heartburn and worsen the condition of peptic ulcer patients	All
Chili	-Irritates the intestinal lining during diarrhoea	All
Fatty foods	-Worsen jaundice	Bari, Sanaag, Sool
Sugary foods like tea, juice, cake	-Increase injury and delay healing of fractures	Nugaal
Fish	-Delays healing of fractures	Mudug
Beans	-Cause infection in people with fractures	Nugaal/Eyl
Mango juice	-Worsens diarrhoea	Mudug
Milk	-Speeds up bowel movements worsening diarrhoea	Mudug, Karkaar
Vegetables	-Speed up bowel movements worsening diarrhoea	Nugaal

## Discussion

### Prevalence of food taboos and demographic characteristics of respondents

88% (88%) of participants in this study reported the existence of food taboos in their communities. This high prevalence in Puntland aligns with broader regional trends. Studies in neighboring East African countries, particularly Ethiopia and Uganda have documented high prevalence rates of food taboos, especially among pregnant women, 67.4% in Ethiopia [21] and 27.9% in Uganda [5]. Elsewhere [2, 10, 22], reported a 64%, 57.8% and 38% prevalence of food taboos among pregnant women in KwaZulu Natal (South Africa), slum settlements in Makassar and Coastal Endenese Indonesia respectively.

Most food restrictions were among women, 32% among pregnant and lactating women and 12% among menstruating women compared to men (20%). This prevalence suggests widespread dietary restrictions, risking nutrient deficiencies especially vitamin A and C, iron, and folate which are vital especially in these vulnerable groups [3, 23].

Participants 40 years and above were found to have much more information regarding food taboos compared to their young counterparts. A community leader in Garowe district asserted the fact that these folks have lived longer in their communities and have accumulated knowledge passed down informally through their ancestors across generations. The younger generation on the other hand is embedded in technology, particularly use of mobile phones, which contribute to evolving cultural and socialization practices, influencing how they learn about and relate to their heritage [24]. During the FGDs and KII, female participants were more actively involved and provided much more information than their male peers provided.

### Food taboos during pregnancy and lactation

Foods mainly restricted for pregnant women are highly nutritious foods like proteins (sheep meat/preserved meat, eggs, liver, kidney and fish). These are very crucial for the rapid growth of the fetus, formation of amniotic fluid, production of hormones and enzymes, enlargement of the uterus and its supporting structures and supporting the increased blood volume needed during pregnancy. Restricting such foods can negatively affect the mother and the unborn child and even contribute to complications after delivery. Avoidance of such foods is reported in different studies. According to [4, 5, 23], pregnant women in Buganda region in Uganda were barred from eating eggs, chicken and pork, associating their consumption with barrenness/infertility and the notion that these are foods for men. In Gambia, pregnant women do not eat eggs for fear of the child becoming dumb, mute or stammer when their speech develops. In Bangladesh, pregnant women do not eat fish, duck meat and mutton, and papaya and pineapples for fear of epilepsy of the child, speech disorders, convulsions, or undesirable traits in the baby and miscarriage respectively [25]. In some regions of Tanzania, pregnant and breastfeeding women were restricted from eating eggs, fish and meat [11].

Consumption of some fruits like papaya, cantaloupe, lemon and vegetables like chili, which are rich in essential micronutrients like vitamins and mineral, are also restricted. This increases the risk for hidden hunger (micronutrient malnutrition) [11]. reported prohibition of pumpkin leaves and cabbage for pregnant women in Tanzania [6], found more anemic pregnant women (26.2%) to adhere to food taboos compared to their non-anemic counterparts (14.6%) in Ethiopia and adherence to food taboos was significantly associated with a higher odds of anemia [2, 6].

### Food taboos during infancy/early childhood

Liver is unanimously prohibited for children less than two years in all regions, associating it with speech delay and that the high concentration of vitamin A would cause health issues to the baby [10, 12]. reported a similar prohibition associated with the baby being born without hair and very active bile causing abscesses in the lungs respectively. Although liver contains high levels of preformed vitamin A (retinol), the amounts and frequency of its consumption in Puntland is limited because liver is a very expensive commodity, with a kilo going for over $18. It is therefore nearly impossible for one to eat it frequently and/or in large amounts that can induce toxicity in this demography [26]. reported vitamin A toxicity to occur as a result of regular ingestion of more than 10,000 IU daily, mainly attributed to synthetic retinoids. Meanwhile [10], found that children less than two years were not allowed to eat ox liver because they would be lazy, stupid and would poop inside the house for the rest of their life. Liver is a good source of vitamins A, which is crucial for fetal growth and development, immune function and good vision. In addition, it is rich in vitamins A, C and B6, minerals iron and cobalamin and proteins, lack of which can lead to a combination of severe health issues, primarily affecting blood formation (anemia), immune function, nerve health, and tissue repair/growth.

Other region specific prohibited foods were fish, eggs, lemon, beans, maize, sorghum, wheat, banana and chili. Other studies done in Nigeria [27], Cape Town - South Africa [3], KwaZulu-Natal - South Africa [10] have reported prohibition of eggs, fish, lemon and beans, bananas and chili, respectively in children less than two years. Our results are contrary to [28] from Lao People’s Democratic Republic, who reported no food prohibitions for children as they eat everything that the rest of the family eats.

### Food taboos among men

Food taboos among adult men have not been widely reported since most researchers evaluate the impact of food restrictions on the categories most vulnerable to malnutrition, specifically young children and women of reproductive age. However, we included men in our study because, in order to combat detrimental food practices, nutrition and health campaigns must understand and engage the men who control household finances and food choices. Our findings reveal the foods mainly considered a taboo for men are generally foods that are considered low quality, including organ meats, head and leg meat, animal heart and neck meat. These are common to all regions and known to be food for women.

Prohibition of these nutrient-dense organ meats (which contain iron, folate, zinc, B Vitamins selenium, collagen/protein and calcium) for men increases their access to women who are more prone to nutritional deficiencies.

Consumption of those foods by men may lead to shame, disrespect, and cowardice, weakening and bending of the man’s neck. In the few countries where men are restricted from eating certain foods, these foods in most cases are not entirely restricted but just specific forms of that food. In Papua New Guinea for example, married young men do not eat fresh meat [12]. They can only eat smoked meat. Men should not also eat flatfish (soles and stingrays), snails and beans as they weaken the warrior’s strength and lead to stomach disorders [12]. Furthermore, according to [13] the Akan men in Nigeria are permanently banned from eating ripe plantain to avoid developing waist pains and taro (a species of cocoyam) else, they will fail to perform sexually. However [29], reported no adherence to any food taboos among men in rural Tajikistan.

### Food taboos during menstruation

To many women, menstruation is a period of discomfort surrounded by cultural beliefs that influence their social behaviors and diets. These diets include taboos aimed at protecting women from perceived health problems or protecting the society against calamity [30]. Foods prohibited in Puntland included cold drinks, milk, coffee, spicy foods and fenugreek [31]. reported cold exposure to be associated with dysmenorrhea as it significantly disrupts blood circulation and prostaglandin production. The same study found the consumption of ice cream during winter to be more associated with dysmenorrhea among Asian women. On the other hand [32–34], found caffeine consumption to be associated with higher intensity of menstrual pain. Caffeine reduces blood flow and forces uterine muscles to work harder, leading to stronger cramps and pain. However, part of our findings are contrary to [35] who reported fenugreek to have anti-inflammatory effects therefore reducing menstrual pain and associated symptoms. They also reported a reduction in duration of pain between two cycles in women who consumed (fenugreek) seeds. In addition, consumption of more dairy products was associated with less menstrual pain and symptoms of dysmenorrhea [36, 37]. Menstrual related food taboos have also been reported in Papua- New Guinea where menstruating women cannot eat bananas and pandanus because eating these fruits would cause the trees to cease to bear fruits [12]. In Haryana- India, milk, buttermilk/curd, potatoes, rice tamarind, sugarcane, pickles, onions, and sugarcane cannot be consumed because they increase pain and cramps [38] and in Malaysia, cooking oils, salt, domestic and wild animals, and monosodium glutamate are restricted because they cause excessive flow of menstrual blood and future ill health [30].

### Food taboos during illness/disease

Some foods are also prohibited among Puntland communities during illnesses like diarrhea, fever and fractures. Foods like chili, mango juice, vegetables and milk cannot be eaten during diarrhea because they are believed to irritate the intestinal lining, speed up bowel movements and worsen the condition. Chili is also believed to cause heartburn and worsen the condition of peptic ulcer patients. These prohibitions have some scientific basis [39, 40]. reported diarrhea-predominant Irritable Bowel Syndrome patients to exhibit gut hypersensitivity to chili, with ingestion producing more abdominal pain and burning in these patients compared to healthy volunteers. In addition [41], reported lactose-free milk products to reduce the risk of worsening diarrhea and reducing the duration of diarrhea by about 18 h. In Nepal, children with diarrhea do not eat spicy and oily foods, hard and cold foods/fluids as they are believed to be harmful [42, 43]. Moreover, breast milk is also believed to be naturally harmful by some Nepalis people and it should be stopped during diarrhea [40, 42, 43].

### Limitations

We used the same FGDs for elderly men and women. Women dominated the discussions and limited men from expressing their views fully because they considered food related subjects to be a women’s issue. Future research should have separate groups for elderly men and women to allow each gender to express their views fully.

Our findings are only on food taboos, their prevalence, people affected and reasons for their existence. Further research should be done to develop effective strategies for delivering nutrition education and challenging the various perspectives held by the communities in culturally sensitive ways. In addition, a study assessing nutritional status should be done to determine if there is a variation in regions like Mudug and Eyl district with more restrictions compared to others.

## Conclusion

Food taboos are common throughout Puntland but they are more rampant in Mudug region and Eyl district of Nugaal region. Some taboos reported during menstruation and illness have some scientific backup and may contribute to better management of these conditions. However, taboos like those reported during pregnancy and lactation may reduce dietary diversity and negatively impact overall nutrition and health outcomes in vulnerable groups.

## Supplementary Information

Supplementary Material 1.

Supplementary Material 2.

Supplementary Material 3.

## Data Availability

All data supporting the findings of this study are available within the paper and its Supplementary Information.

## Abbreviations

FGD: Focused Group Discussion
FSNAU: Food Security and Nutrition Analysis Unit
IBS: Irritable Bowel Syndrome
IPC: Integrated Food Security Phase Classification
KII: Key Informant Interview
KI: Key Informant
UNICEF: United Nations Children’s Fund
SPSS: Statistical Package for Social Sciences
