# Supplementary material for: Exploring life cycle and health-related food taboos and their associated reasons among Puntland - Somalia communities: a mixed methods cross sectional study
Source: BMC Nutr. 2026 Jul 9;12:206. doi: 10.1186/s40795-026-01413-1 (PMC13640307; doi:10.1186/s40795-026-01413-1)
Supplement: Supplementary file 1 [file 40795_2026_1413_MOESM1_ESM.docx]

**QUESTIONNAIRE ON FOOD TABOOS**

**SECTION 1: DEMOGRAPHIC CHARACTERISTICS OF RESPONDENTS**

1. **Gender**
   A. Male
   B. Female

**2. Age of respondent**

A. 20-30 years
B. 30-40 years
C. 40-50 years
D. More than 50 years

**3. Marital status**

A. Married
B. Singe
C. Widowed
D. Divorced

**4. Education level**

A. Informal education
B. Primary level
C. Secondary level
D. College/university

**SECTION 2: PREVALENCE OF FOOD TABOOS**

**5. Are you aware of any prevalent food taboos among the people of your region?**

A. Yes
B. No

**6. During which life stages/condition are food taboos most strictly observed?**

B. Pregnancy and lactation
C. Infancy and childhood
D. Menstruation
E. Illness
F. Adulthood (Men)

**14. Reasons for prohibition**

A. They don’t recover
B. An infection develops in the bone

**15. When a person have a gallbladder ill**

A. Ghee and sheep fat
B. oil foods
C. meat
D. spices

**16. Reasons for prohibition**

A. They believe they will not heal
B. They believe an infection is developing
C. They believe they cannot digest food

**17. When someone is taking medicine**

A. ghee
B. lemon
C. beans

**18. Reasons for prohibition**

A. They believe the medicine will not work if they take it.
B. They believe an infection is developing

**19. Food prohibited for girls during ministration**

A. Chilli
B. Cold water

**20. Reasons for prohibition**

A. They believe their throat is closing
B. They believe an infection is developing
