# Supplementary material for: Exploring life cycle and health-related food taboos and their associated reasons among Puntland - Somalia communities: a mixed methods cross sectional study
Source: BMC Nutr. 2026 Jul 9;12:206. doi: 10.1186/s40795-026-01413-1 (PMC13640307; doi:10.1186/s40795-026-01413-1)
Supplement: Supplementary file 2 [file 40795_2026_1413_MOESM2_ESM.docx]

**QUESTIONS FOR KEY INFORMANTS**

Please tell us your age, education level, position, and work experience, [record gender as observed].

1. From your professional or leadership role and experience, what are the most common foods or food groups that people in this community are restricted from eating, or advised to avoid? Probe each group [pregnancy, lactation, infancy/ early childhood, adult men, illness, or menstruation] until saturation.
2. What are the sources of these restrictions? (e.g., Religion, tradition, elderly/family, health beliefs, or fear of a negative outcome).
3. What are the specific reasons or beliefs behind avoiding those foods? Probe each group [pregnancy, lactation, early childhood, young and adult men, illness, or menstruation] until saturation.
4. What are the perceived negative consequences of consuming a taboo food?
5. How strictly are these taboos followed?
6. What happens if someone breaks a taboo? (e.g., social shaming, family conflict, minor illness, etc.)
