# Supplementary material for: Exploring life cycle and health-related food taboos and their associated reasons among Puntland - Somalia communities: a mixed methods cross sectional study
Source: BMC Nutr. 2026 Jul 9;12:206. doi: 10.1186/s40795-026-01413-1 (PMC13640307; doi:10.1186/s40795-026-01413-1)
Supplement: Supplementary file 3 [file 40795_2026_1413_MOESM3_ESM.docx]

**FOCUS GROUP DISCUSSION INTERVIEW GUIDE**

A participant should state their number whenever they respond to a question or provide contributions during the discussion. Probe each stage until saturation.

**Infancy/Childhood**

1. Which foods are forbidden for children under 5 years, and why?
2. Are there differences in taboos for boys versus girls?

**Pregnancy**

1. What foods do pregnant women avoid?
2. What benefits or risks do you associate with following these restrictions?

**Lactating**

1. During breastfeeding, which foods or drinks are avoided for the mother's or baby's health?
2. How do these practices change over the lactation period?

**Adulthood/Men**

1. Do men have any food restrictions?
2. Which foods are restricted and why?
3. How strictly are these rules followed, and what happens if broken?

**During Illnesses**

1. When someone is sick (e.g., fever, diarrhea, injured etc), which foods are prohibited?
2. Why are these foods prohibited?

**Menstruation**

1. What foods must menstruating women avoid, and for how many days?
2. Are there beliefs about how certain foods affect menstrual flow or future health?
